# Early Life Stress Delays Sexual Maturation in Female Mice

**DOI:** 10.3389/fnmol.2019.00027

**Published:** 2019-02-26

**Authors:** Gabriela Manzano Nieves, Arielle Schilit Nitenson, Hye-In Lee, Meghan Gallo, Zachary Aguilar, Angelica Johnsen, Marilyn Bravo, Kevin G. Bath

**Affiliations:** ^1^Department of Neuroscience, Brown University, Providence, RI, United States; ^2^Department of Cognitive, Linguistic and Psychological Sciences, Brown University, Providence, RI, United States

**Keywords:** sexual maturation, estrous cycle, early life stress, anxiety, development, limited bedding

## Abstract

In humans, some forms of early life stress (ELS) have been linked with precocious puberty, altered brain maturation, and increased risk for a variety of forms of pathology. Interestingly, not all forms of ELS have been found to equally impact these metrics of maturation. In recent work, we have found that ELS in the form of limited bedding (LB) from P4 to P11, was associated with precocious hippocampus maturation in males and increased risk for depressive-like pathology and attentional disturbance in female mice. Here, we sought to test whether ELS in the form of LB also impacted the timing of sexual maturation in female mice. To establish rate of somatic and sexual development, distinct cohorts of mice were tested for weight gain, timing of vaginal opening, and development of estrous cycling. ELS animals weighed significantly less than controls at every timepoint measured. Onset of vaginal opening was tracked from P21 to 40, and ELS was found to significantly delay the onset of vaginal opening. To test the impact of ELS on estrous cycle duration and regularity, vaginal cytology was assessed in independent groups of animals using either a continuous sampling (daily from P40 to P57) or random sampling approach (single swab at P35, P50, or P75). ELS did impact measures of estrous cycling, but these effects were dependent upon the sampling method used. We also tested the impact of ELS on anxiety-like behaviors over development and across the estrous cycle. We observed a developmental increase in anxiety-like behavior in control but not ELS mice. No effect of estrous cycle stage was found on anxiety-like behavior for either group of mice. Together these results provide evidence that ELS in the form of LB delays somatic and sexual development. Additional work will be required to determine the mechanism by which ELS impacts these measures, and if these effects are common to other models of ELS in rodents.

## Introduction

In humans, a variety of forms of early life stress (ELS), ranging from famine to physical abuse, have been associated with effects on the timing of sexual maturation. A bulk of these studies have found that ELS is associated with precocious menarche (Moffitt et al., 1992; Ellis and Garber, 2000; Chisholm et al., 2005; Belsky et al., 2015). Precocious sexual development has been linked to increased risk for susceptibility to physical pathology, including increased lifetime risk for breast and reproductive cancers and for emotional pathology, including increased risk for anxiety and depressive disorders (Kelsey et al., 1993; McPherson et al., 1996; Marshall et al., 1998; Collaborative Group on Hormonal Factors in Breast Cancer, 2012; Bodicoat et al., 2014). The relationship between the timing of sexual maturation and mental health outcomes have been posited to be related to a mismatch between real and perceived age of females at puberty (Caspi and Moffitt, 1991; Ge et al., 1996; Dick et al., 2000; Graber, 2013; Mendle et al., 2017). Understanding the environmental factors that impact the timing of sexual maturation, and associated change in risk for pathology holds great potential for both public policy and strategies for intervention.

Multiple life history theories have been proposed to account for effects of ELS on the timing of sexual maturation, including the predictive adaptive response (Gluckman and Hanson, 2004), energetics theory (Frisch, 1990) and the psychosocial acceleration theory (Mishra et al., 2009). In the predictive adaptive response theory, the quality of care giving serves as a signal to the developing organism about the quality of the external environment (Ellis and Del Giudice, 2014; Belsky et al., 2015; Shalev and Belsky, 2016). These signals serve to shape development by impacting the timing of somatic, neural, and behavioral maturation. In this model, ELS can serve as a signal of a low resource or dangerous environment and drive adaptive changes in the timing of reproductive maturation (e.g., delays in resource poor environments and acceleration in potentially dangerous environments). In the psychosocial acceleration theory, it is argued that high levels of stress should lead to accelerated sexual maturation. In this model, a harsh environment is a signal of increased risk of mortality, and by proxy, decreased longevity of the organism. One proposed evolutionary strategy to promote survival of the species is to promote earlier reproduction (Belsky et al., 1991). In support of this model, multiple published reports have found accelerated sexual maturation in females to be associated with prenatal stress, troubled family relations, mothers with mood disorders, higher allostatic load, and an absent father (Moffitt et al., 1992; Graber et al., 1995; Mezzich et al., 1997; Kim and Smith, 1998; Ellis and Garber, 2000; Allsworth et al., 2005; Chisholm et al., 2005; Costello et al., 2007; Belsky et al., 2015). As a counterpoint to the psychosocial acceleration theory, the energetics theory proposes that energy availability is the key determinant for the timing of sexual maturation (Frisch, 1990). As such, reduced access to nutrition or effects on metabolic function leading to low body weight or lower energy availability, should result in delayed sexual development. Thus, ELS in the form of food insecurity, poor parental care, or resource restriction may favor a delay in both somatic and sexual maturation. Support for this has come from work associating higher SES, lower marital discord, and greater parental support with decreased body mass and later sexual maturation (Ellis and Essex, 2007). In more recent work, children that were previously institutionalized children (PI) and subsequently adopted into United States households were studied. Investigators found that PI youth did not demonstrate the anticipated acceleration in pubertal development that may have been expected with exposure to high levels of ELS, and instead did not differ from control populations (Reid et al., 2017). Thus, not all forms of disruption in the quality or quantity of early life care will have uniform effects on the timing of sexual development. Alternatively, in this group, adoption into affluent or high care households was sufficient to prevent precocious pubertal-onset due to high SES or greater parental investment. Based upon the variety of types of ELS encountered, and differing effects on the timing of sexual maturation, we sought to determine whether a mouse model of ELS, in the form of limited bedding (LB), would alter the timing of sexual maturation.

Use of a rodent model allowed for the dissection of the somatic and physiological consequences of ELS in the form of LB (Harrison and Baune, 2014; Bath et al., 2017b; Walker et al., 2017) including direct effects on sexual maturation and secondary effects on the development of anxiety-like behaviors. Previous work in other models of ELS on sexual development in rodents, including altered licking and grooming or maternal separation, have found changes in copulatory behavior and effects on the timing of vaginal opening, but no effects on the weight of sexual organs in rats (Lau et al., 1996).

Here, we found that ELS in the form of LB led to delays in vaginal opening and decreased somatic weight. Furthermore, ELS was associated with changes in the time spent in different phases of the estrous cycle, but these effects were dependent upon the sampling method employed (continuous or random sampling). Assessing the effects of ELS on the development of anxiety-like behavior, an effect of age on anxiety-like behavior was found for control but not ELS mice. Further, no effect of estrous cycle phase was found for either group of mice. Together, the current findings add to our understanding of the effects of differing forms of ELS on the timing of sexual development. The current results also open up a number of questions regarding the mechanisms supporting changes in sexual development, what metrics should be used to track development, how they are sampled, and how these factors may impact neurobehavioral outcomes.

## Materials and Methods

### Subjects

**Figure 1:**
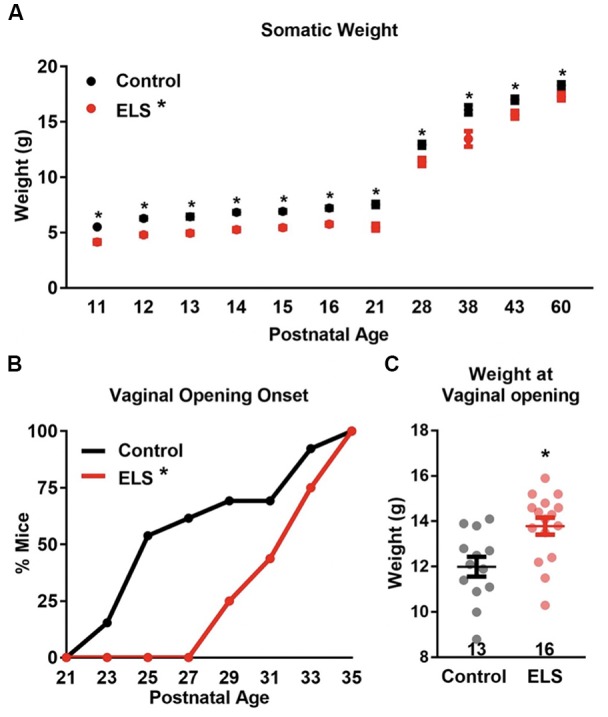
Early life stress (ELS) decreases body weight and delays vaginal opening onset. **(A)** ELS females weigh consistently less than control females during development and into adulthood. Dots represent group means ± SEM (CR females, *n* = 12–34 per age; ELS females, *n* = 5–29 per age). **(B)** ELS delays vaginal opening onset in female mice. Graph represents the cumulative percentage of mice who had vaginal opening onsets by a given age. **(C)** The weight at which vaginal opening onset occurs was greater for ELS females than controls. *N* of the groups for panels **(B,C)** are presented in panel **(C)**. A two-way analyses of variance (ANOVA) followed by a *post hoc* multiple comparison test **(A)**, Log-rank **(B)**, and a two-tailed unpaired *t*-test **(C)** were used to assess statistical significance between groups *p < 0.05.

**Figure 2:**
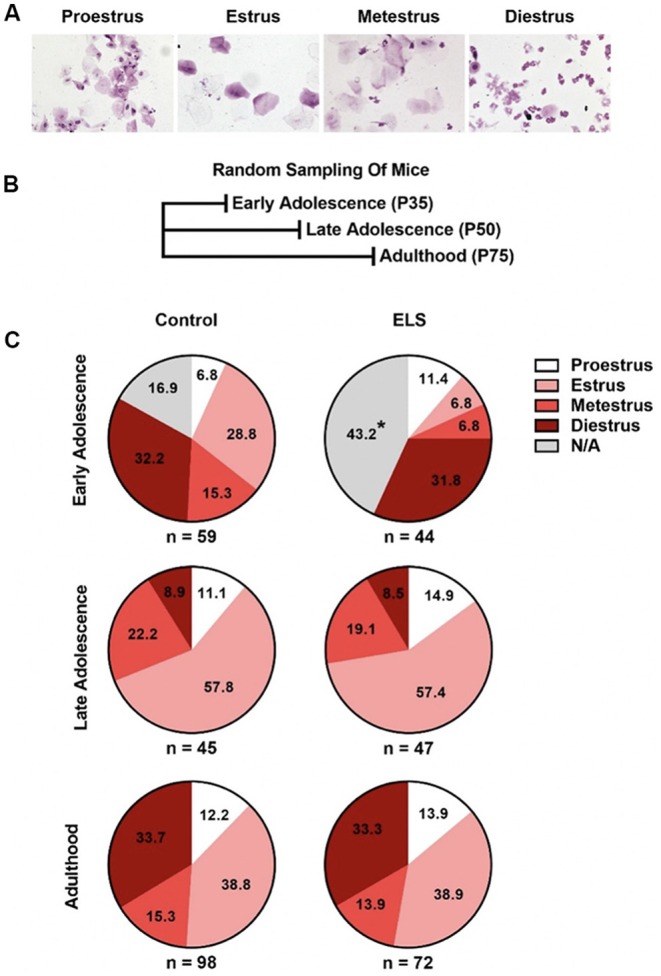
ELS delays complete vaginal openings but does not affect the probability of being at a given estrous cycle. **(A)** Example pictures of the estrous cycle phases. **(B)** Schematic of random sampling protocol. Distinct cohorts of mice were used at each sampled age. **(C)** ELS early adolescent mice have a greater portion of females without complete vaginal openings and trend toward significant differences in the distribution of estrous cycle phases (top). ELS does not alter the distribution of females in a given estrous cycle phase during late adolescence (middle) or adulthood (bottom). Pie charts represent the portion of females in a given estrous cycle phase. *N* of the group is presented underneath each pie chart. Chi-squared test were used to assess statistical significance between groups *p < 0.05.

**Figure 3:**
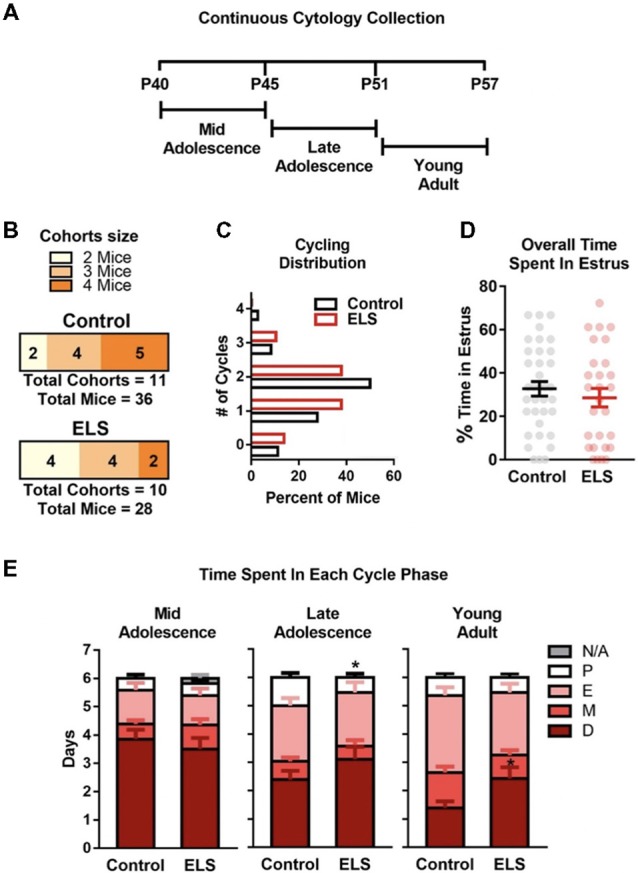
ELS does not alter estrous cycling in adolescence but may affect length of cycle phases. **(A)** Schematic of continuous sampling protocol. Daily vaginal smears were obtained from postnatal day 40 to 57. **(B)** Description of total mice in each group and the number of mice in each cohort.** (C)** Graph depicting the percent of mice that were cycling. ELS did not alter the distribution or number of estrous cycles **(D)** or the overall time spent in estrous during the 18-day protocol (P40–57). **(E)** However, ELS did decrease the cumulative number of days mice spent in proestrus during late adolescence (P46–51). Furthermore, ELS increased the cumulative number of days spent in diestrus during young adulthood (P52–57). Lines and bars represent group means ± SEM. Dots in **(D)** represent individual values. The number of mice in each group is shown in panel **(B)**. Chi-square test was used to assess differences in distribution **(C)**. Unpaired two-tailed student *t*-test were used to assess statistical significance between groups *p < 0.05.

**Figure 4:**
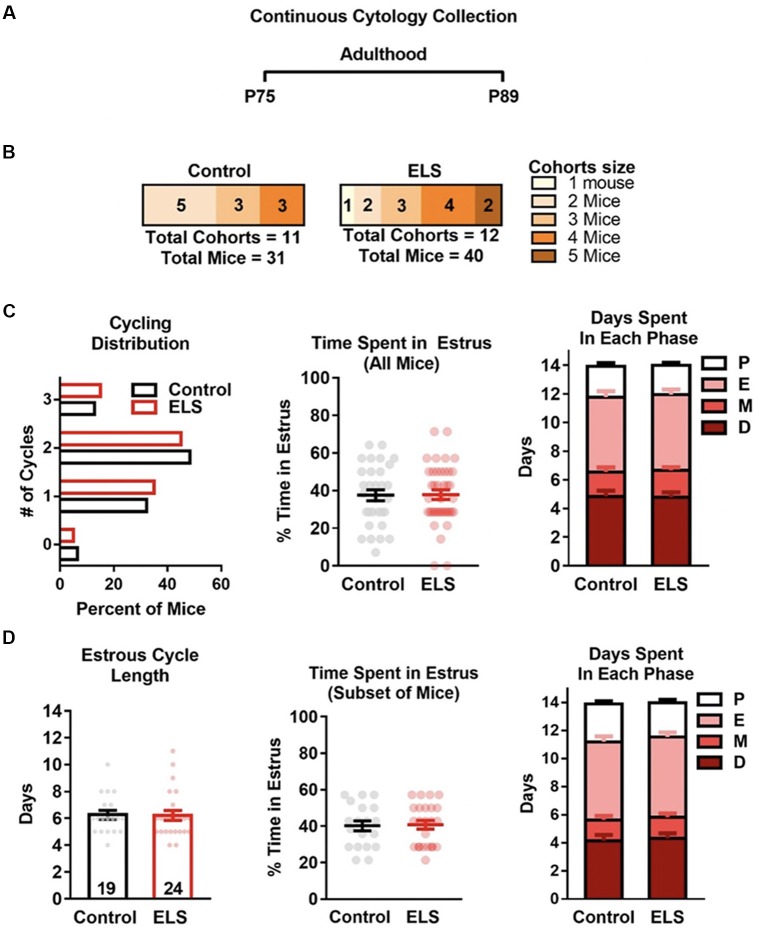
ELS does not affect estrous cycling in adulthood. **(A)** Schematic of continuous sampling protocol. Daily vaginal smears were obtained from postnatal day 75 to 89. **(B)** Description of total mice in each group and the number of mice in each cohort.** (C)** Graph depicting the percent of mice that were cycling. ELS did not alter the distribution or mean number of estrous cycles (left), or the total time spent in the estrous cycle during the 14-day protocol (center). Furthermore, ELS did not change the cumulative number of days mice spent in each cycle phase (right). **(D)** Graphs depicting the effects of ELS in the subset of mice that had two or more cycles during the 14-day protocol. ELS did not alter the length of the estrous cycle (left), the time spent in estrus (center), nor the time spent in any of the other phases (right). Bars represent group means ± SEM. Dots represent individual values. The total *n* for graphs in **(C)** is depicted in panel **(B)**. The *n* of graphs in panel **(D)** are presented on the bars of (**D** left). Chi-square test was used to assess differences in distribution (**C** left); unpaired two-tailed student *t*-test were used to assess statistical significance between groups *p < 0.05.

**Figure 5:**
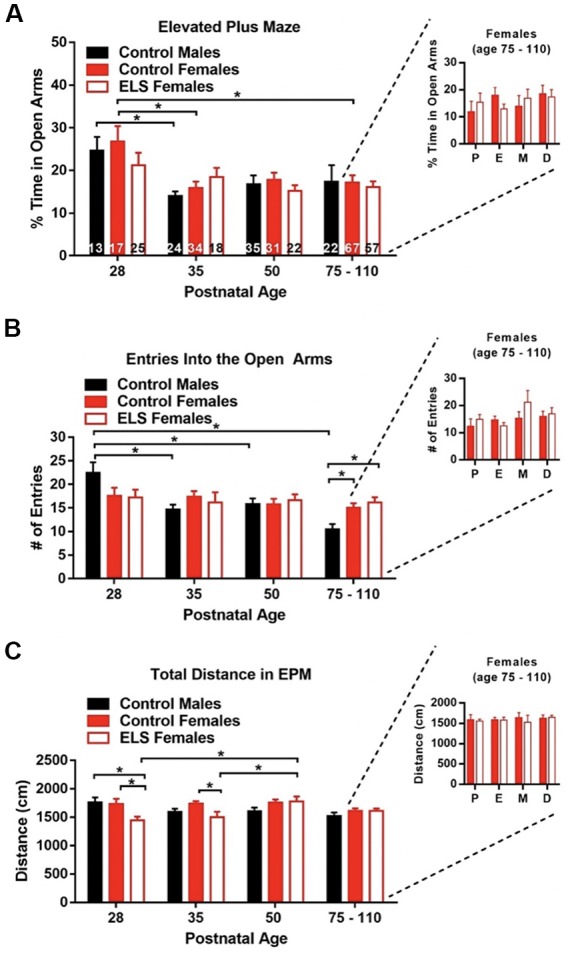
Age only affects anxiety outcomes. **(A)** Time spent in the open arms (anxiogenic arms) of the elevated plus maze (EPM) decreases with age. This effect seems to be mainly driven by control animals. ELS does not affect the time spent in the open arms at any age. Estrous cycle phase was not seen to alter the time spent in the open arms in adult mice (right inset). **(B)** The number of entries into the open arms decreased with age for control males but not control or ELS females. ELS did not affect the entries into the open arms at any age when compared to control females. Estrous cycle phase was not seen to alter entries into the open arms in adult mice (right inset). **(C)** ELS decreased the distance walked for females at P28 and P35 when compared to control females. Estrous cycle phase was not seen to distance traveled in the EPM in adult mice (right inset). Bars represent group means ± SEM, the *n* of each group of the main figures are shown on the bars of panel **(A)**. Control females in the insets = 67, ELS females in the insets = 52. A two-way ANOVA followed by a *post hoc* multiple comparison test was used to assess statistical significance between and within groups *p < 0.05.

A total of 550 C57BL/6N virgin mice were used for this study. Subjects were sex segregated following weaning at age (P21). For any given cohort, multiple litters were sampled from (~3–6 litters) to eliminate potential cohort effects. Cohorts ranged in size with 1–8 females per cohort. Testing of singly housed mice was limited. Example distribution of mice cohorts are shown in Figures 3B, 4B. All animals were housed on a 12:12 h light:dark cycle and had *ad libitum* access to food and water throughout the study. Mice cages were housed in mixed female/male cage holding racks. Twenty-nine mice were used for Figures 1A,B. Sixty-five mice (36 control and 29 ELS) were sample across various days for Figure 1C, the exact number of mice sampled for a given age are shown in Supplementary Table S1. A total of 261 female and 94 male mice were used in Figure 5. Following the elevated plus maze (EPM) test, a subset of these mice was used for the random (Figure 2) and continuous sampling to assess cycling (Figures 3, 4). To achieve sufficient sample size for analysis, an additional 101 mice were sampled in Figure 2. A subset of the mice in Figure 2 were continuously sampled for Figures 3, 4. All animal procedures were approved by the Brown University Institutional Animal Care and Use Committee and were consistent with the National Institutes of Health Guide for the Care and Use of Laboratory Animals.

### Limited Bedding (LB)

ELS was induced by providing LB material to dams when pups were between the ages of P4 to P11. This model induces a fragmentation in maternal care, where the dam exhibits a greater number of nest exits and entries compared with dams that have access to the full complement of bedding materials (Rice et al., 2008; Bath et al., 2016). Specifically, 4 days after the birth of a litter (P4), the dam and pups were transferred from their standard home cage with cob bedding and a 4 × 4 cm cotton nestlet to a cage containing a wire mesh floor and only a 3 × 4 cm cotton nestlet. The mice continued to have *ad libitum* access to food and water. Following 1 week (at P11), pups and dams were returned to their standard housing. The control reared mice (controls) were left undisturbed in a standard home cage during this period.

### Vaginal Opening

To determine the onset of vaginal opening, two independent observers inspected the vaginal opening of mice for the visual appearance of an opening starting at postnatal day 21 and ending at postnatal day 45. To minimize stress and discomfort of mice, the vaginal status was assessed every other day. Upon visual observation of vaginal opening, status was verified by passing a small cotton swab along the outer opening of the vagina. The appearance of any opening was defined as the onset of vaginal opening. In Figure 1, the age and weight at which a vaginal opening onset was first detected was noted. For experiments in which vaginal smears were collected, a closed or partially open vaginal canal, which were insufficient for complete penetration for swabbing, resulted in a not available (N/A) designation. Vaginal smear collection required the vaginal canal to be completely open for samples to be collected. The number or proportion of mice from which smears could not be collected on a given day are indicated in each figure.

### Estrous Cycle Monitoring

Estrous cycle was monitored using two different approaches. In a subset of mice, a random sampling approach was used in which vaginal smears were collected from mice at a single timepoint (postnatal day 35, 40, 50, or 75). The random sampling approach eliminated potential stimulatory effects, or effects of stress associated with repeated vaginal swabbing on cycle onset. In a separate cohort of mice, a repeated sampling (continuous) approach was used. For continuous sampling, vaginal smears were collected for either 18 (starting at P40) or 14 (starting at P75) consecutive days between the hours of 5 pm to 7 pm. All mice used for this study were previously unhandled.

Estrous cycle stage was determined by vaginal cytology. Vaginal cytology was assessed by dipping a sterile swab in water and gently swabbing the outer half of the vaginal canal. Vaginal samples were transferred to a microscope slide, air dried, stained using a Hema 3 staining kit (Fisher Scientific, Hampton, NH, USA), dehydrated, and then cover slipped prior to visualization on a light microscope at 10× magnification. Estrous cycle stage was assessed using previously established criteria (Bath et al., 2008; Aliagas et al., 2010; Byers et al., 2012) and were carried out by an observer blind to rearing condition and behavioral results. Vaginal smears were not collected from mice that did not have complete vaginal openings, these mice are labeled not available (N/A) in relevant figures. For explanation see “Vaginal Opening” section.

The number of cycles was defined as the number of times that proestrus or Diestrus preceded estrus. Whereas the length of the estrus cycle was analyzed as the number of days between proestrus to the next proestrus, or the number of days between proestrus to an estrus that directly followed Diestrus. In the example: P E M D P E M D E M D there are three cycles with the first cycle lasting 4 days. Only the length of the first complete cycle was analyzed for this study.

### Elevated Plus Maze

To assess anxiety-like behavior during development, mice were tested on an EPM at either P28, P35, P50, P75–110. Mice were tested only once on this apparatus and an individual mouse contributed to only a single time point. The EPM apparatus was built in-house and consisted of two open (unprotected) and two closed (protected) arms, previously described (Manzano-Nieves et al., 2018). Greater time in the closed (protected) arms is defined as higher anxiety-like behavior while increasing time in the open arms is indicative of a lower anxiety-like state.

A trial began by placing a mouse in the center of the arena and allowing it to explore the maze for 7 min. The time spent in the protected vs. unprotected arms was assessed. Videos were recorded, and behavior was tracked using Noldus Ethovision XT 10.0 software. All trials were conducted under low light conditions (~109 Lux). Time spent, visits and distance walked in the open and closed arms of the EPM were assessed using the mouse tracking module. Trials in which a mouse fell off the arena were discarded (6 out of 130 at P75), and the mouse was removed from the experiment. Within an hour of completing the behavioral test, vaginal cytology samples were obtained. Behavioral testing occurred between the hours of 3–7 pm, shortly before lights off in the housing facility.

### Data Analysis

Data was analyzed using Prism software (Prism, GraphPad Software, La Jolla, CA, USA) and SPSS (IBM). Graphs and images were made using GraphPad Software. Differences in distributions were assessed using a Chi-square test. Differences in onset of vaginal openings was assesses using a Log-rank test. A two-way analyses of variance (ANOVA) followed by Sidak’s multiple comparison test was used to assess differences in weight (Figure 1A). Multiple two-tailed unpaired *t*-tests with a Holm-Sidak multiple comparison correction were used for comparing the number of days spent in each cycle stage in Figures 3E, 4C right, and 4D right. All other *t*-tests used were also unpaired and two-tailed. In Figure 5, two-way ANOVAS followed by *post hoc* Tukey tests were used to assess differences between groups.

## Results

### Early Life Stress Delay Somatic Development

To determine the effect of ELS on somatic and sexual maturation, whole body weight and the timing of vaginal opening were measured. Body weight of control and ELS mice were measured across development (postnatal ages: 11, 12, 13, 14, 15, 16, 21, 28, 38, 43, and 60) Figure 1A. A main effect of age (two-way ANOVA- *F*_(11,367)_ = 689.6, *p* < 0.0001) was found with both groups gaining weight across development. However, weight between the groups differed (*F*_(1,367)_ = 195, *p* < 0.0001), with ELS mice weighing less than controls at all time points measured. No interaction between rearing condition and age were found (*F*_(11,367)_ = 1.27, *p* = 0.25). A subsequent Sidak’s multiple comparisons *post hoc* test comparison revealed that ELS mice weighed significantly less than controls at every age tested (see Supplementary Table S1).

Assessment of vaginal opening was determined by sampling animals on alternating days from postnatal day 21 to postnatal day 35. The appearance of any size vaginal fissure constituted the onset of vaginal opening. Vaginal openings were first observed in control mice at P23 and in ELS mice the first appearance of vaginal opening was observed at P29 (Figure 1B). A Log-rank test revealed that control females underwent vaginal openings at a younger age than ELS females (${Χ}_{1,N=29}^{2}$ = 5.779, *p* = 0.0162), suggesting that ELS females experience delayed somatic sexual development. Furthermore, the mean age at vaginal opening onset was significantly greater for ELS mice compared with controls (*t*-test: Control = 27.62 vs. ELS = 32.25; *t*_(27)_ = 3.64, *p* = 0.0011).

Diminished body weight has been shown to correlate with delayed or suppressed sexual maturation in humans (Frisch and McArthur, 1974; Vigersky et al., 1977; Warren, 1980). It is possible that lower body weights in ELS mice significantly contributed to the delay in vaginal openings. If the decrease in weight was driving the delay in vaginal opening, then it would be predicted that ELS mice should demonstrate vaginal opening at the same weight in which control mice exhibited vaginal opening. Surprisingly, the weight of ELS mice at the onset of vaginal opening was significantly greater than the mean weight of control mice at vaginal opening (*t*-test: *t*_(27)_ = 3.13, *p* = 0.0042; Figure 1C).

### Early Life Stress Delays Onset of Estrous Cycling

To determine if ELS impacts the onset of estrous cycling, we used a random sampling (Figure 2B) approach. Representative images of vaginal cytology for each of the four estrous cycle stages are shown in Figure 2A. Random sampling of estrous stage allowed us to assess the distribution of cycle phase without the potential confounds of handling, repeated vaginal stimulation, or stress on this measure. For random sampling, cohorts were sampled a single time at either postnatal day 35, 50, or 75. The proportion of mice in each stage of the estrous cycle is presented for each age assessed (Figure 2). Chi square tests were performed to assess the effects of ELS on the proportion of mice in each stage of the estrous cycle.

During early adolescence (P35), ELS mice differed significantly from controls in the proportion of mice with complete vaginal openings (${Χ}_{1,N=103}^{2}$ = 8.57, *p* = 0.0034; Figure 2C top). Approximately 43% of ELS mice, but only 17% of control reared mice, did not have complete vaginal openings. As mice with incomplete vaginal openings could not be swabbed for cycle assessment, this decreased the number of mice from which vaginal smears could be obtained in the ELS group, hindering our ability to test for differences in cycle phase between the two groups. When mice without complete vaginal opening were excluded from the analysis, no differences between the groups were observed (${Χ}_{3,N=74}^{2}$ = 6.58, *p* = 0.087). However, this failure to detect a significant difference may have been the result of needing to exclude a large percentage of ELS female mice that did not have complete vaginal opening at P35 (43%). Given the resultant small sample size, the exact effect of ELS at this timepoint remains unclear. To determine if the differences observed in early adolescence continued into adulthood, separate cohorts of mice were randomly sampled for estrous status at either P50 (late adolescence) or P75 (adults). Chi-square analysis of the distribution at P50 (${Χ}_{3,N=92}^{2}$ = 0.36, *p* = 0.95; Figure 2B middle) and P75 (${Χ}_{3,N=170}^{2}$ = 0.14, *p* = 0.99; Figure 2B bottom) indicated that rearing condition did not impact the distribution of cycle phases observed in those cohorts of mice.

### ELS Alters the Periodicity of Estrous Cycling in Adolescence

The estrous cycle length shortens and becomes more regular as mice transition from young adulthood to full adulthood (approx. P120; Nelson et al., 1982). However, whether ELS impacts the development of a more regular and shorter cycle remains unknown. To test if ELS altered the number or length of estrous cycles, a continuous monitoring method was employed. Estrous cycle was tracked by daily collection of vaginal smears. To assess ELS effects on cycling during adolescence into young adulthood (P40–57; Figure 3), vaginal smears were collected for 18 consecutive days starting at P40. The total number of mice and the number of mice per cohort used are shown in Figure 3B. As adolescence represents a dynamic developmental stage, the data was binned into 6-day blocks (Figure 3E) to enable visualization of changes in the days spent in each cycle phase.

During development (P40–P57), no differences in the overall distribution (${Χ}_{4}^{2}$ = 2.007, *p* = 0.73) or mean value of estrous cycles were observed (*t*_(62)_ = 0.94, *p* = 0.35; Figure 3C). Furthermore, ELS did not impact the overall percent of time that mice spent in the estrus stage of the estrous cycle (*t*_(62)_ = 0.78, *p* = 0.44; Figure 3D). During mid adolescence (P40–45; Figure 3D left), ELS and control mice spent approximately the same number of days in proestrus (*t*_(62)_ = 0.064, *p* = 0.59), estrus (*t*_(62)_ = 0.43, *p* = 0.59), metestrus (*t*_(62)_ = 1.40, *p* = 0.59), and diestrus (*t*_(62)_ = 0.70, *p* = 0.59). Furthermore, there was no differences in the number of days that mice spent without complete vaginal openings (N/A; *t*_(62)_ = 1.60, *p* = 0.11). During late adolescence (P46–51; Figure 3D middle) ELS mice were found to spend less time in proestrus than controls (*t*_(62)_ = 2.16, *p* = 0.034). ELS did not affect the time spent in any of the other estrous cycle phases; estrus (*t*_(62)_ = 0.18, *p* = 0.86), metestrus (*t*_(62)_ = 0.71, *p* = 0.48), or diestrus (*t*_(62)_ = 1.36, *p* = 0.18). During young adulthood (P52–57; Figure 3D right) there was a significant difference in the amount of time ELS mice spent in diestrus (*t*_(62)_ = 2.39, *p* = 0.020) compared to controls, with ELS spending on average more time in diestrus. ELS did not alter the time in proestrus (*t*_(62)_ = 0.56, *p* = 0.58), estrus (*t*_(62)_ = 1.19, *p* = 0.24), or metestrus (*t*_(62)_ = 1.49, *p* = 0.14) during young adulthood.

### ELS Rearing Does Not Affect Estrous Cycle Into Adulthood

To determine if ELS effects the estrous cycle of adult mice, the number of estrous cycles, estrus duration, and the time spent in each estrous stage were assessed. For assessing differences during adulthood (P75–89; Figure 4), vaginal smears were collected for 14 consecutive days starting at P75. As binning P75 data did not reveal effects of time only overall (14-day) data was analyzed and discussed.

To determine if there were differences in the estrous cycle of adult mice reared under control or ELS conditions, vaginal smears were collected daily from mice as detailed in Figure 4A. As the number of mice per cohort can alter cycling, the number of mice per cohort are presented in Figure 4B. The number and average length of the estrous cycles was compared for ELS and control reared mice. A *t*-test revealed that ELS rearing did not affect the distribution (${Χ}_{3}^{2}$ = 0.20, *p* = 0.98; Figure 4C left), mean number (Control mean = 1.68 vs. ElS mean = 1.7; *t*_(69)_ = 0.12, *p* = 0.91) or the mean time mice spent in estrus (*t*_(69)_ = 0.082, *p* = 0.93; Figure 4C center). An analysis of the number of days spent in each of the four estrous cycle phases revealed no significant differences between ELS and controls during proestrus (*t*_(69)_ = 0.43, *p* = 0.67), estrus (*t*_(69)_ = 0.14, *p* = 0.89), metestrus (*t*_(69)_ = 0.45, *p* = 0.65), or diestrus (*t*_(69)_ = 0.12, *p* = 0.91; Figure 4C right). However, it is possible that our analysis is being skewed or masked by the high number of mice with irregular estrous cycles (defined as less than two cycles; see Figure 4C left). Therefore, we assessed possible cycling differences within the portion of mice that underwent two or more estrous cycles (Control = 61%, ELS = 60%; Figure 4D). We found that ELS mice did not significantly differ from control mice in the length of the estrous cycle (*t*_(41)_ = 0.11, *p* = 0.91; Figure 4D left) or in the number of days spent in estrous (*t*_(41)_ = 0.16, *p* = 0.87; Figure 4D center). Furthermore, an analysis of the number of days spent in each of the four estrous cycle phases, for the subset of mice that were regularly cycling, revealed no significant differences between ELS and controls (proestrus: *t*_(41)_ = 0.28, *p* = 0.89; estrus: *t*_(41)_ = 0.50, *p* = 0.98; metestrus: *t*_(41)_ = 0.39, *p* = 0.98; diestrus: *t*_(41)_ = 0.53, *p* = 0.98; Figure 4D right).

### Age, but Not ELS Rearing or Estrous Cycle Stage, Impacts Anxiety-Like Behavior

Previous work has shown that estrous cycle stage can have a significant effect on the expression of anxiety-like behavior in mice (Gangitano et al., 2009; Bath et al., 2012). While ELS does impact the onset of vaginal opening and possibly the onset of stable estrous cycling, it does not appear to impact the duration of estrous cycling in adulthood (Figure 4). To determine if ELS rearing impacted the expression of anxiety-like behavior in mice, we used the EPM. Independent groups of mice were sampled across development, and estrous cycle phase was determined for each animal immediately following testing. A two-way ANOVA revealed a main effect of age on time spent in the open arms (*F*_(3,353)_ = 7.88, *p* < 0.0001), with decreasing levels of open arm exploration as mice aged, suggesting that anxiety-like behavior increases with age. To determine what times points might be driving the main effect of age, we carried up follow-on *post hoc* testing collapsing across rearing condition. We found that at P28, mice spent significant more time in the open arms when compared to P35 (*p* = 0.0012), P50 (*p* = 0.0034), and P75 (*p* = 0.0012) mice. However, given that not all groups had the same *n* (Figure 5A), and that the effect of age may not be equal across all groups, we performed a *post hoc* Tukey to assess within group differences across ages. No main effect of rearing condition (*F*_(2,353)_ = 0.52, *p* = 0.60) or interaction between rearing condition and age (*F*_(6,353)_ = 0.71, *p* = 0.64) were found, suggesting that ELS rearing did not significantly affect anxiety-like behavior. To assess what ages where contributing to the main effect of age on anxiety-like behavior, we performed a *post hoc* Tukey test assessing the main effect was conducted. We found, after collapsing across conditions, that P28 mice spent significant more time in the open arms when compared to P35 (*p* = 0.0012), P50 (*p* = 0.0034), and P75 (*p* = 0.0012) mice. However, given that not all groups had the same *n* (Figure 5A), and that the effect of age may not be equal across all groups, we performed a *post hoc* Tukey to assess within group differences across ages (Supplementary Table S2). We found that control females spent a greater percentage of time in the open arms at P28 than at P35 (*p* = 0.012) and P75–110 (*p* = 0.016), and a trend towards more time in the open arms at P28 when compared to P50 (*p* = 0.061). For control males a difference between the percent time spent in the open arms was reported for P28 vs. P35 (*p* = 0.046), with P28 control animals spending more time in the open arms. In *post hoc* Tukey multiple comparison tests, no effects of sex or rearing condition were found at any of the ages tested (Supplementary Table S3). Additionally, no effects of the estrous cycle on anxiety like behavior were observed in adulthood (P75–110; Figure 5A right inset). A two-way ANOVA revealed that neither estrous cycle phase (*F*_(3,111)_ = 0.51, *p* = 0.68) nor rearing condition (*F*_(1,111)_ = 9.27 × 10^−8^, *p* = 0.9998) effected the percent time females spent in the open arms. Furthermore, no interaction between estrous cycle and rearing condition was observed (*F*_(3,111)_ = 0.51, *p* = 0.60).

We also assessed rearing effects on percent entries into the open arms in the EPM. A two-way ANOVA revealed a main effect of age (*F*_(3,353)_ = 6.021, *p* = 0.0005) and interaction between group and age (*F*_(6,353)_ = 2.78, *p* = 0.012), suggesting that entries into the open arms decreased with increasing age of mice tested. No main effect of group was found (*F*_(2,353)_ = 0.24, *p* = 0.79). *post hoc* Tukey multiple comparison tests were used to assess simple effects of age and group. Significant age differences were only observed within control males, which had significantly more entries at P28 than at any other time point (all *p* < 0.05). Control males also had significantly more entries at P50 than they did at P75–110. Furthermore, at P75–110 control males had decreased entries into the open arms when compared to control females (*p* = 0.038) and ELS females (*p* = 0.0081). This data suggests that the number of entries decreases with age in control males, but not control or ELS females. Furthermore, no effects of the estrous cycle on the number of entries into the open arms were observed in adulthood (P75–110; Figure 5B right inset). A two-way ANOVA revealed that neither estrous cycle phase (*F*_(3,111)_ = 1.85, *p* = 0.14) nor rearing condition (*F*_(1,111)_ = 1.11, *p* = 0.29) effected the number of entries into the open arms. Additionally, no interaction between estrous cycle and rearing condition was observed (*F*_(3,111)_ = 1.016, *p* = 0.39).

To determine if any of the observed effect of age, or failures to observe treatment or sex effects could be attributed to primary effects on locomotion, we tested for effects of these variables on the distance traveled by mice in the EPM. A two-way ANOVA revealed a main effect of group (*F*_(2,353)_ = 3.92, *p* = 0.021) and interaction between group and age (*F*_(6,353)_ = 3.12, *p* = 0.0055), suggesting that total distance walked in the EPM differed between groups. However, no main effect of age was found (*F*_(3,353)_ = 2.20, *p* = 0.088). A *post hoc* Tukey multiple comparison test was used to assess the differences between groups at a given age. At P28 we found that ELS females walked less than control males (*p* = 0.013) and control females (*p* = 0.014) of the same age. The difference between the distance walked persisted in P35, with control females walking significantly more than ELS females (*p* = 0.032). Only ELS females displayed age effects on distance walked. ELS females walked significantly more at P50 than at P28 (*p* = 0.0029) or P35 (*p* = 0.037). Interestingly, these changes in locomotion did not impact the number of entries or the percent time in the open arms. Further experiments will be needed to fully understand the impact of ELS on locomotor behavior during early adolescence and adolescence. To assess if the estrous cycle was impacting locomotion, we assessed the distance traveled in the EPM for control and ELS females in adulthood (P75–110; Figure 5C right inset). A two-way ANOVA revealed that neither estrous cycle phase (*F*_(3,111)_ = 0.19, *p* = 0.90) nor rearing condition (*F*_(1,111)_ = 0.25, *p* = 0.61) effected the number of entries into the open arms. Additionally, no interaction between estrous cycle and rearing condition was observed (*F*_(3,111)_ = 0.18, *p* = 0.91).

## Discussion

Here, we assessed the impact of ELS in the form of LB on the timing of sexual maturation in female mice. ELS led to delays in vaginal opening, decreased body weight, and transient effects on estrous cycling. Time spent in different phases of the estrous cycle were largely restricted to the early adolescent period and unaltered during late adolescence and early adulthood. Interestingly, no effect of estrous cycle stage was found on anxiety-like behavior in control or ELS females. In fact, only increasing age was found to be associated with an increase in anxiety-like behavior. Together our findings add to the current literature and provide evidence that ELS in the form of LB delay aspects of sexual maturation in a mouse model.

To assess the effect of ELS on somatic development we tracked weight gain and the onset and completion of vaginal opening. We found that ELS delayed weight gain and the onset (Figure 1B) and completion (Figure 2C) of vaginal opening. This is in contrast with findings from a maternal separation paradigm, where investigators found an acceleration in the onset of vaginal opening (Grassi-Oliveira et al., 2016), but no effect on the completion of vaginal opening (Rhees et al., 2001; Grassi-Oliveira et al., 2016). The disparities between these models may be related to differences in the form of stress encountered during development and what they signal to the developing organism. In the psychosocial acceleration theory, the repeated loss of the parent may signal either instability or greater probability of the loss of a caregiver, and thus the need to mature faster, exit the nest sooner, and possibly reproduce earlier. In the current work, limited access to bedding does not impact contact time between the dam and the pup or the total amount of care provided. However, this form of stress does impact weight gain of the pups, possibly through decreased nutrition provided by the dam, or effects on basal stress hormones levels, metabolism, or thermoregulation. Thus, this model may better approximate effects expected from the energetics theory of maturation, where decreased resources may elicit a delay in aspects of somatic maturation. Alternatively, the shift in sexual maturation that was observed could be interpreted in the context of the predictive adaptive response theory. It is possible that stress associated with the decrease in availability of resources may be promoting a delay in sexual development as a means of delaying reproduction in a low resource environment. This may provide advantages in the context of drought and famine when resources for caring for young are not available and provide a means to delay reproduction until such times pass. However, additional experiments altering the home-cage environment post ELS rearing would be necessary to test this hypothesis. The link between delay in vaginal opening and decreased body weight has previously been reported by various groups in pre-pubertal maternal separation in rodents (McIntosh et al., 1999; de Almeida Magalhães et al., 2017). Interestingly, studies in post-institutionalized children have reported similar decreases in body weight and no effect of institutional rearing on physical signs of puberty (Walker et al., 2007; Hayes and Tan, 2016; Reid et al., 2017). It is possible that environmental changes associated with adoption at a very young age may be sufficient to buffer against delays in sexual development, or that these effects may be mirroring an energetics model of reproductive development. Together these results suggest that ELS in the form of LB induces reduced weight gain over development, which may delay somatic signs of sexual development in a dose dependent manner. Studies comparing differing type, intensity, and length of ELS will be needed to completely parse out the relationship between these variables.

Over the course of development, there are significant changes in the regularity and duration of the estrous cycle in mice. As mice transition from late adolescence to adulthood, the cycle becomes shorter and more regular (Nelson et al., 1982). To test if ELS altered the amount of time mice spent in different phases of the estrous cycle during adolescence and young adulthood, we used two different approaches in two separate groups of mice, a cross-sectional (random sampling) and longitudinal (repeated sampling) approach. In the cross-sectional group (Figure 2), there was a trend for ELS effects on the distribution of mice in a given estrous phase during early adolescence (*p* = 0.087). However, this group suffered from under sampling due to many ELS mice (43%) not having complete vaginal openings. A larger sample size may have allowed us to view significant difference in the estrous phase distribution of ELS mice in early adolescence. In our continuously sampled group, we did not find any effects of ELS during the early adolescent period (Figure 3). It is possible that the effects observed in the cross-sectional group were missed in our continuously sampled group because of differences in the ages sampled. Whereas the cross-sectional group was sampled at P35 the longitudinal was first sampled at P40. However, the continuous sampling group, did reveal effects of decreased proestrus during late adolescence and increased diestrus in young adulthood. Because these effects only occurred in the continuously sampled group of mice, it is possible that continuous sampling acted as a secondary stressor. Therefore, the later effects observed in the continuously sampled data could have been a result of a condition by sampling method interaction.

In humans, anxiety disorders first emerge during pre-adolescence, with the median age of onset at 11 years of age (Kessler et al., 2005). Stress incurred early in life can increase the risk for developing anxiety-related behaviors (Agid et al., 1999; Draijer and Langeland, 1999; Widom, 1999; Heim and Nemeroff, 2001; Koenen and Widom, 2009). Curiously, our lab and others have found that ELS in the form of LBN is not associated with increased expression of anxiety-like behaviors in adulthood (Molet et al., 2016; Goodwill et al., 2018; Manzano-Nieves et al., 2018). However, a failure to exhibit an anxiety-like phenotype as an adult does not preclude the possibility that ELS impacts the expression of anxiety-like behavior over development (e.g., possible transient developmental expression of anxiety-like behaviors that may resolve by adulthood). To determine if ELS impacted the development of anxiety-like behavior, different groups of mice were tested once at either P28, P35, P50 or P75+. Rearing condition did not affect anxiety-like behavior at any of the ages tested. This both replicates and expands upon our previous findings, suggesting that a low resource model of ELS does not increase the propensity for anxiety-like behavior in a mouse model. However, we did observe an overall main effect of age on levels of anxiety-like behavior, whereby P28 mice showed significantly less anxiety than P35, P50 or P75+ mice. The decrease in anxiety at P28 may be reflective of a bias toward an increase in exploratory behavior at this age (Bath et al., 2017a). Decreasing anxiety and increasing exploration at P28 may be advantageous under non-stress conditions, as it promotes independence from dams and the discovery of new resources in the wild. Interestingly, ELS reared females specifically did not display an increase in anxiety-like behavior over development. These results may indicate that ELS is associated with an earlier elevation in anxiety-like behavior in female mice. Additional data from younger cohorts of mice would be required to directly test this hypothesis. Given that the variability of males and females are known to be explained by different facets of behavior in the EPM (Fernandes et al., 1999), assessing anxiety-like behavior in different behavioral paradigms (light/dark box or open field) will be necessary to conclusively determine the impacts of ELS and development on anxiety.

Together, the data presented here led us to conclude that ELS in the form of LB in females resulted in decreased weight gain and delayed sexual maturation, as indexed by the onset of vaginal opening. The effects of ELS on weight gain are consistent with our previous work in males (Bath et al., 2016). It is possible that the effects of ELS on weight gain, may be driving the observed delays in vaginal opening. Prior work has found that body weight may serve as a better predictor of puberty onset than chronological age (Kennedy and Mitra, 1963; Frisch, 1972), possibly due to effects on leptin levels, a hormone produced by adipose cells, that has been shown to be necessary for both puberty and fertility (Ahima et al., 1997; Yura et al., 2000; Farooqi, 2002; Smith et al., 2002). To directly test such a hypothesis, work manipulating both ELS and restoring body weight, through manipulations such as high fat diet, would be required. Future work will be needed to test this hypothesis.

## Author Contributions

GMN and KB conceived the project and designed the experiments. GMN performed behavioral experiments with the aid of H-IL. GMN and MG performed the “Vaginal Opening” section analysis experiment. GMN and ASN analyzed vaginal cytology. GMN obtained continuous vaginal smears with the aid of ZA, AJ and MB. GMN interpreted data, wrote and edited the manuscript with the aid of KB.

## Conflict of Interest Statement

The authors declare that the research was conducted in the absence of any commercial or financial relationships that could be construed as a potential conflict of interest.
