# Supplementary material for: Early Life Stress Delays Sexual Maturation in Female Mice
Source: Front Mol Neurosci. 2019 Feb 26;12:27. doi: 10.3389/fnmol.2019.00027 (PMC6399387; doi:10.3389/fnmol.2019.00027)
Supplement: Supplementary file 1 [file Table_1.DOCX]

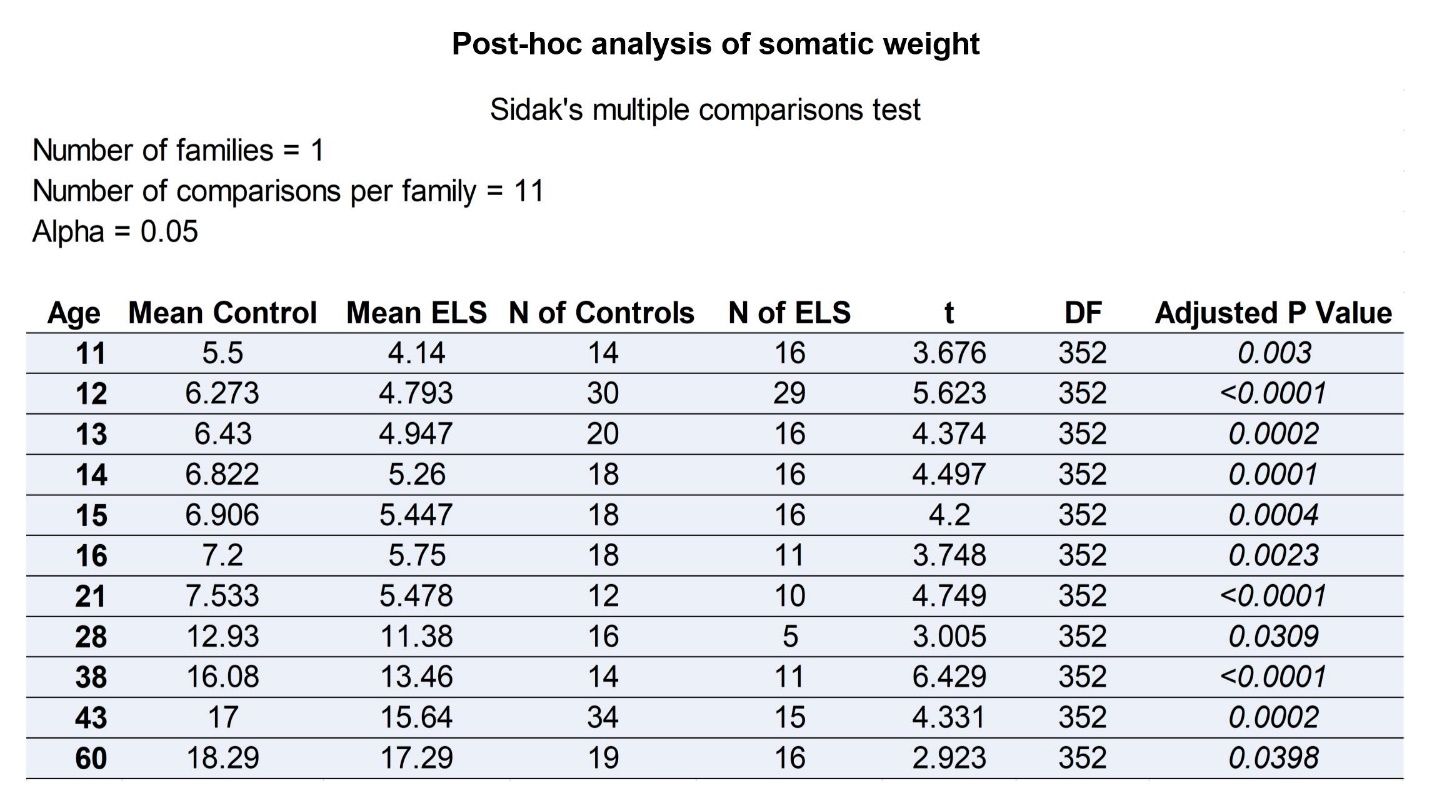


**Supplementary Table 1.** Statistics for post-hoc analysis of whole-body weight during development, presented in Figure 1A.


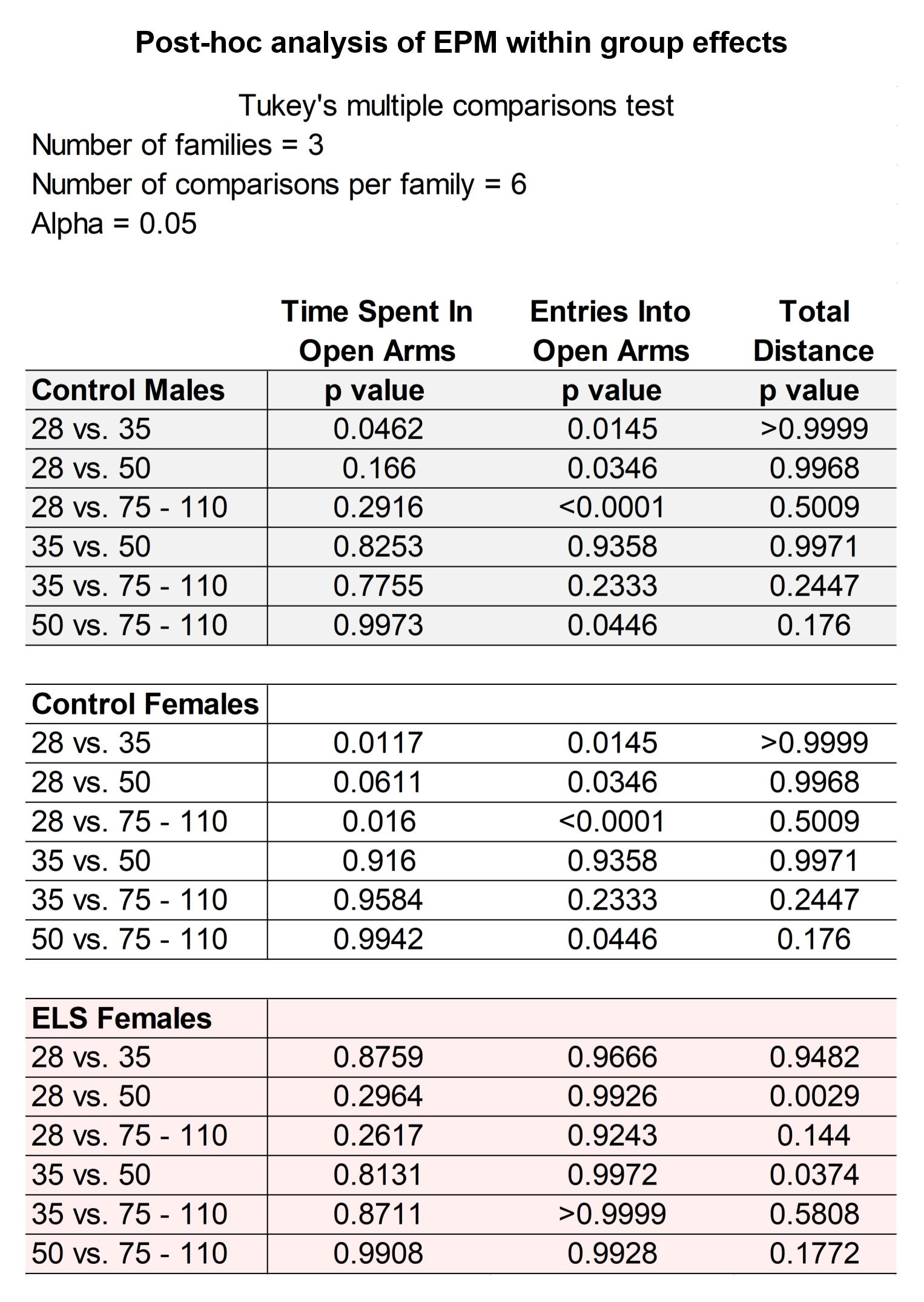


**Supplementary Table 2.** Statistics for Tukey’s post-hoc within group analysis for data shown in Figure 5. Table summarizes the adjusted p-value outputs of three distinct post-hoc tests: time in open arms, entries into open arms and, total distance.


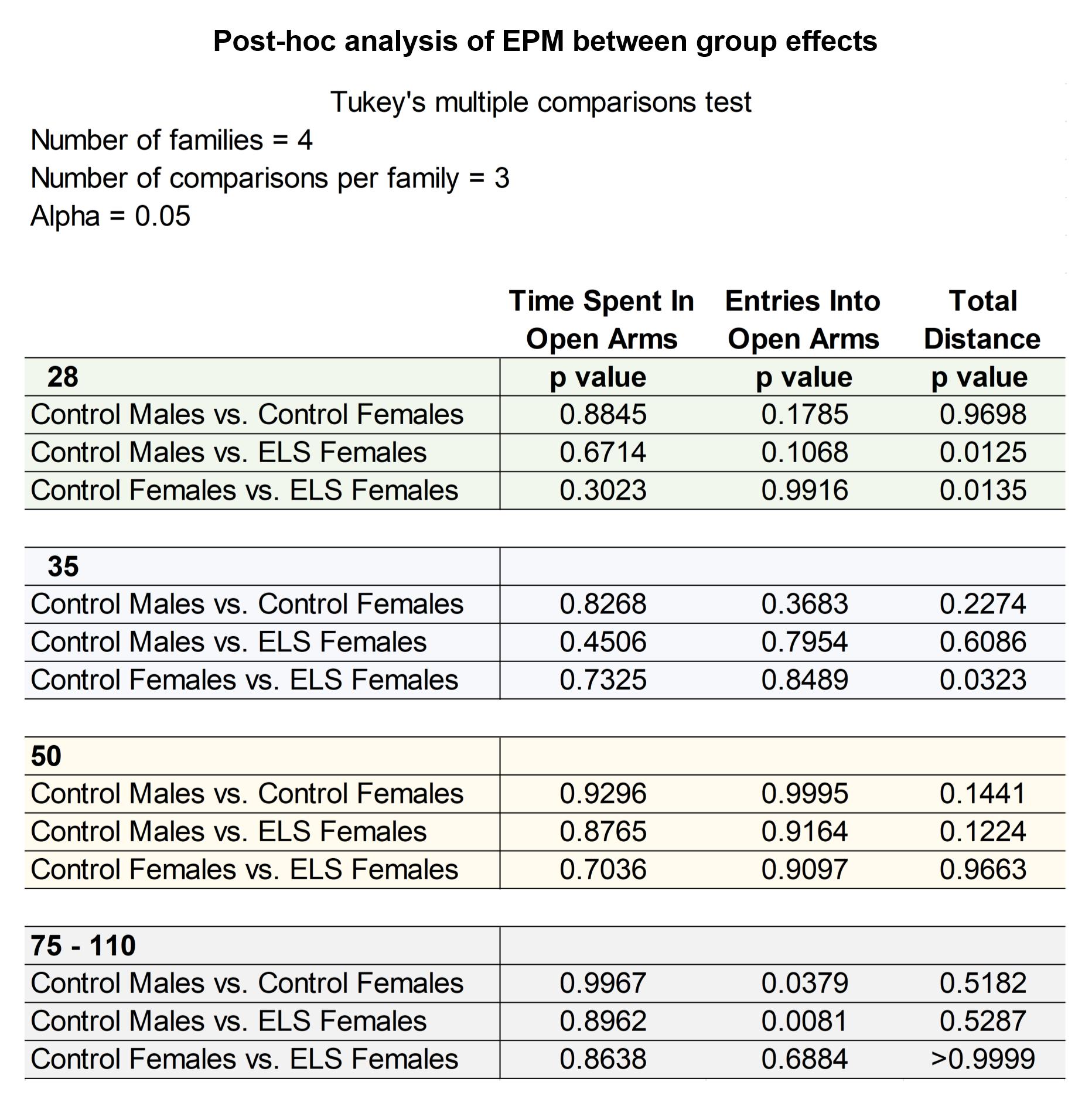


**Supplementary Table 3.** Statistics for Tukey’s post-hoc between group analysis for data shown in Figure 5. Table summarizes the adjusted p-value outputs of three distinct post-hoc tests: time in open arms, entries into open arms and, total distance.
